# A case of subacute combined degeneration of the spinal cord diagnosed with difficulty due to a history of treatment for pyogenic spondylitis

**DOI:** 10.1002/ccr3.7180

**Published:** 2023-04-07

**Authors:** Yoshiki Shimokawa, Yuki Ishikawa, Teiki Okawa, Mana Higashihara, Fumiaki Tokimura, Tsuyoshi Miyazaki, Kentaro Hayakawa

**Affiliations:** ^1^ Department of Orthopaedics and Spine Surgery Tokyo Metropolitan Institute for Geriatrics and Gerontology Tokyo Japan; ^2^ Department of General Medicine Tokyo Metropolitan Institute for Geriatrics and Gerontology Tokyo Japan; ^3^ Department of Neurology Tokyo Metropolitan Institute for Geriatrics and Gerontology Tokyo Japan

**Keywords:** atrophic gastritis, gait disturbance, pyogenic spondylitis, subacute combined degeneration, vitamin B12

## Abstract

Early diagnosis of spinal cord subacute combined degeneration (SCD) is difficult, especially in pre‐existing lower extremity impairment cases. We report a case of progressive SCD diagnosed after severe anemia. The peripheral symptoms of SCD other than gait disturbance should also be well understood and given close attention.

## Graphical abstract

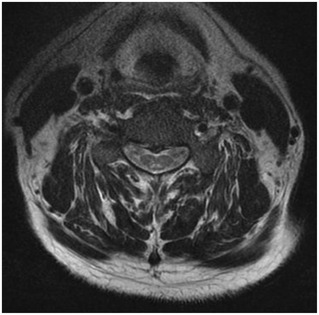

## 1 INTRODUCTION

Subacute combined degeneration (SCD) of the spinal cord is a neurological complication associated with a deficiency of vitamin B12, which is necessary for the development and initial myelination of the central nervous system.^1^ Limb numbness, limb weakness, and gait disturbances are common symptoms in patients with SCD.^2^ In a magnetic resonance imaging (MRI) study of the spinal cord, the T2 hyperintense signal within the posterior funiculus on axial images is distinctive.^3^, ^4^ Patients often present with hematological, gastrointestinal, psychiatric, and neurological symptoms.^5^ Early diagnosis and treatment of SCD are essential because the damage to the nervous system is often irreversible, and the progression of other peripheral symptoms, such as anemia, is life‐threatening. However, the diagnosis of SCD is sometimes challenging because of its variable clinical presentations.

Here, we report a case of progressive SCD diagnosed after severe anemia. This patient had a gait disturbance before the onset of SCD because of a previous history of pyogenic spondylitis.

## 2 CASE HISTORY/EXAMINATION

**FIGURE 1:**
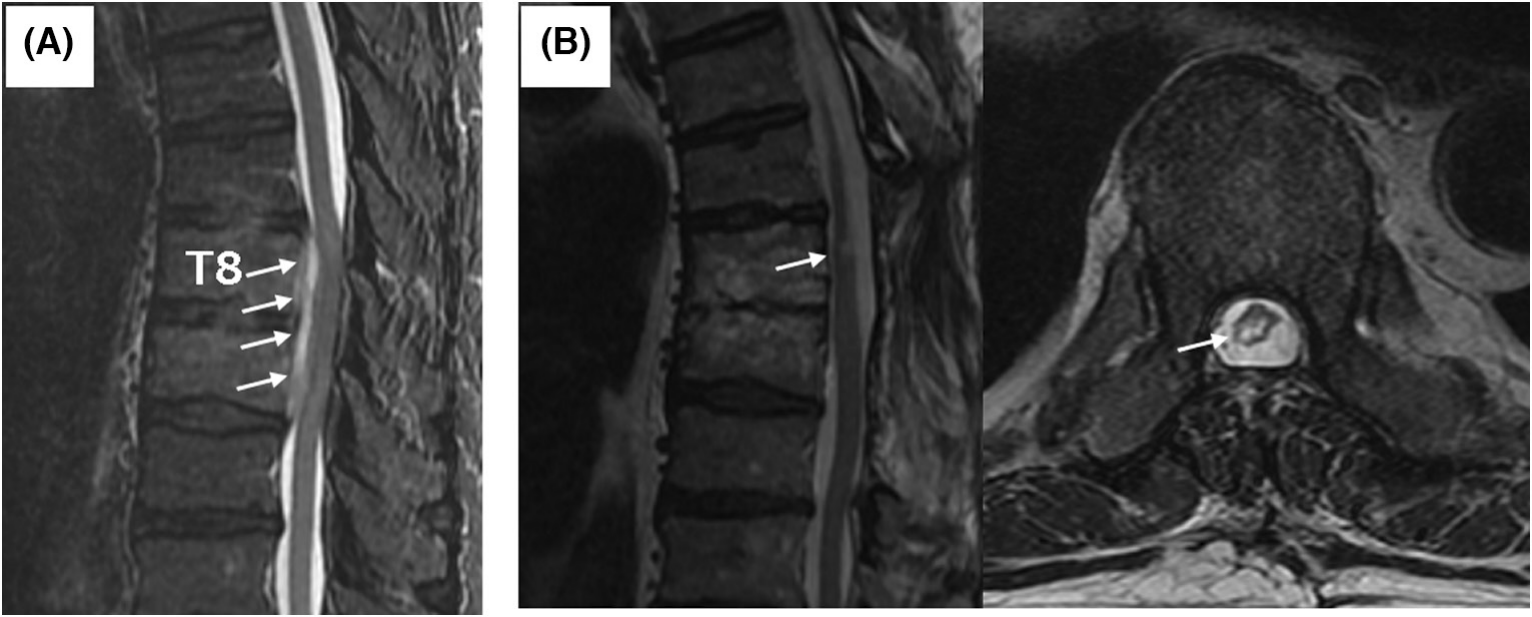
Magnetic resonance imaging of the thoracic spine before. (A) and 6 months after (B) treatment with pyogenic spondylitis. (A) Sagittal T2WI showing signal changes in the eighth and ninth thoracic vertebral body and epidural abscess (white arrows) compressing the spinal cord from the ventral side. (B) Epidural abscess and compression of the spinal cord were resolved, yet hyperintense signal change (white arrows) remains in sagittal and axial T2WI.

A 66‐year‐old Japanese man with numbness in both hands underwent emergency posterior decompression surgery 1 year and 6 months before presentation for paralysis of both legs because of pyogenic spondylitis and epidural abscess of the thoracic spine (Figure 1A). He was prescribed antibiotics for 6 months. The laboratory data improved after treatment started and the infection was considered cured (Table 1). Leg paralysis gradually improved over the weeks after surgery, although he sometimes used a walking cane because of residual gait disturbance. However, hyperintense signal changes in the spinal cord on T2‐weighted thoracic MRI remained after resolution of the pyogenic spondylitis and epidural abscess (Figure 1B).

**TABLE 1:** Laboratory data during the treatment for the pyogenic spondylitis.

Items (unit)	Measured value			Reference value range in our hospital
	Preoperative	2 weeks postoperative	2 months postoperative	
White blood cell count (/μL)	7800	5700	5600	3300–8600
Hemoglobin (g/dL)	12.6	11.8	12.8	13.7–16.8
C‐reactive protein (mg/dL)	11.49	5.82	<0.30	<0.30

He had no other surgical history, including a gastrectomy. He had been diagnosed with atrophic gastritis at his annual medical checkup, but it was not considered necessary to treat it.

The numbness of hands gradually worsened, and general fatigue appeared 4 months after the appearance of numbness of the hands, although he could walk with a cane, as before. Neurological examination showed enhancement of deep tendon reflexes in the lower extremities, which was the same as before. Touch and vibratory sensation were not decreased. Romberg's sign and Babinski's sign were not present.

**FIGURE 2:**
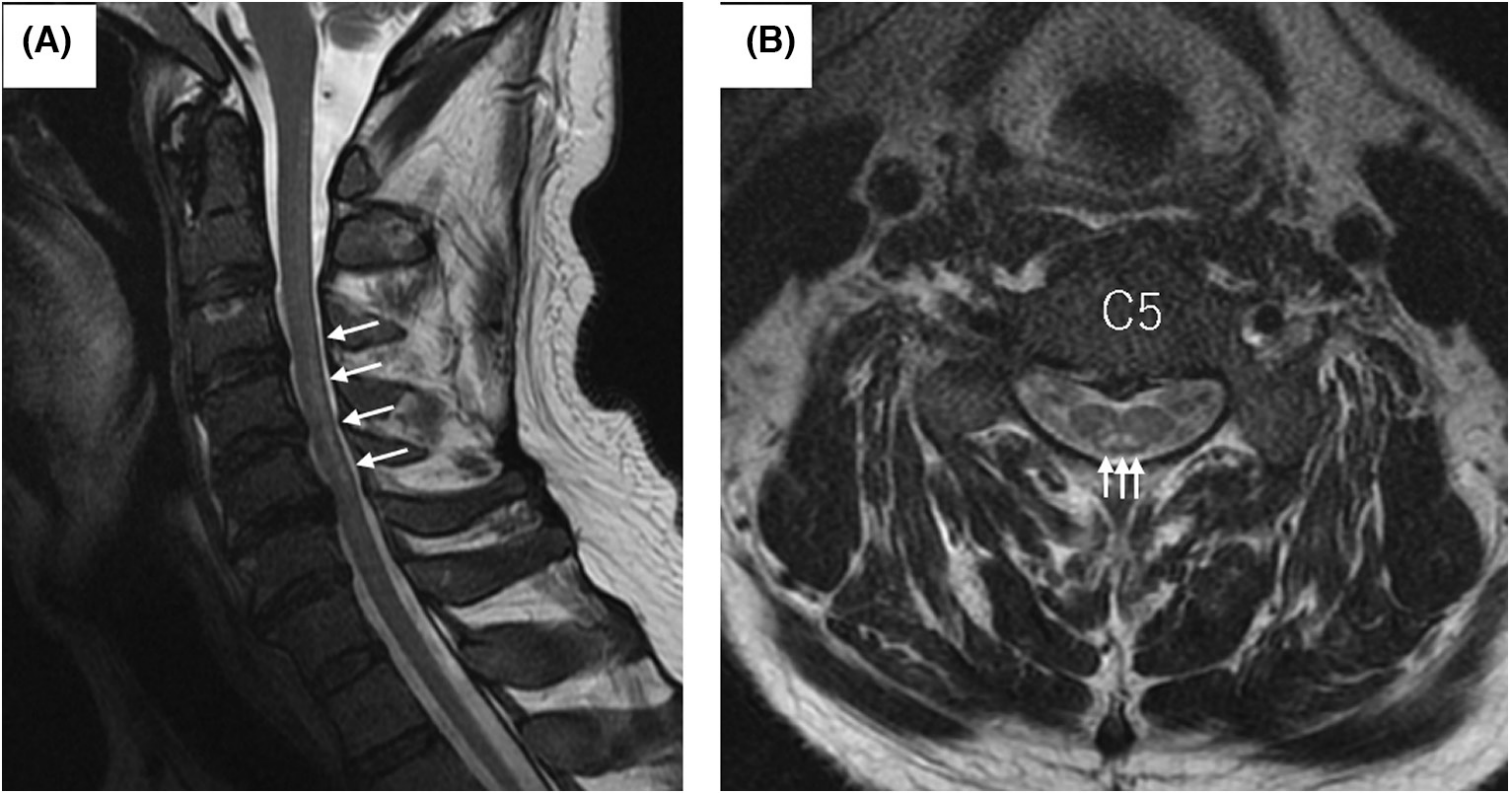
Magnetic resonance imaging of the cervical spine before treatment with subacute combined degeneration. (A) Sagittal T2WI showing longitudinally extensive hyperintense signal changes (white arrows) within the spinal cord. (B) Axial T2WI showing a bilateral hyperintense signal within the posterior funiculus, demonstrating the “inverted V‐sign” (white arrows).

MRI of the cervical spine demonstrated hyperintensity in the dorsal columns (appearing as an inverted V sign) at C3‐C6 on T2‐weighted imaging (Figure 2), consistent with SCD of the spinal cord. Laboratory tests performed immediately after the complaint of fatigue showed severe megaloblastic anemia (hemoglobin levels, 4.8 g/dL) and a low serum vitamin B12 (or cobalamin) level (71 pg/mL). Serum folic acid and copper level were not decreased (Table 2). Nerve conduction study showed no evidence of peripheral neuropathy, while somatosensory evoked potentials from the tibial nerve demonstrated prolonged central conduction time consistent with involvement of central somatosensory pathways in the spinal cord. Further, motor‐evoked potentials showed prolonged central motor conduction time, indicating involvement of pyramidal tracts.

**TABLE 2:** Laboratory data of the blood before treatment of subacute combined degeneration.

Items (unit)	Measured value	Reference value range in our hospital
White blood cell count (/μL)	4790	3300–8600
Red blood cell count (×10^4^/μL)	124	435–555
Hemoglobin (g/dL)	4.8	13.7–16.8
Mean corpuscular value (fL)	116.1	83.6–98.2
Mean corpuscular hemoglobin (pg)	38.7	27.5–33.2
Platelet count (×10^4^/μL)	10.7	15.8–34.8
Vitamin B12 (pg/mL)	71	233–914
Folic acid (ng/mL)	13.8	3.6–12.9
Copper (μg/dL)	98	70–132

The patient was treated with blood transfusion on the day of admission. Additionally, the patient was treated intravenously with cyanocobalamin 1000 μg daily for the first week and intramuscularly with cyanocobalamin 500 μg three times a week for 3 months.

## 3 OUTCOME AND FOLLOW‐UP

His general fatigue improved shortly after blood transfusion and cyanocobalamin administration. No additional transfusion was needed while he regularly continued cyanocobalamin administration intramuscularly after the discharge.

With regard to neurological symptoms, numbness in both hands also improved gradually over the months, although it remained slight. Gait disturbance resided, but he was able to walk without a cane.

**FIGURE 3:**
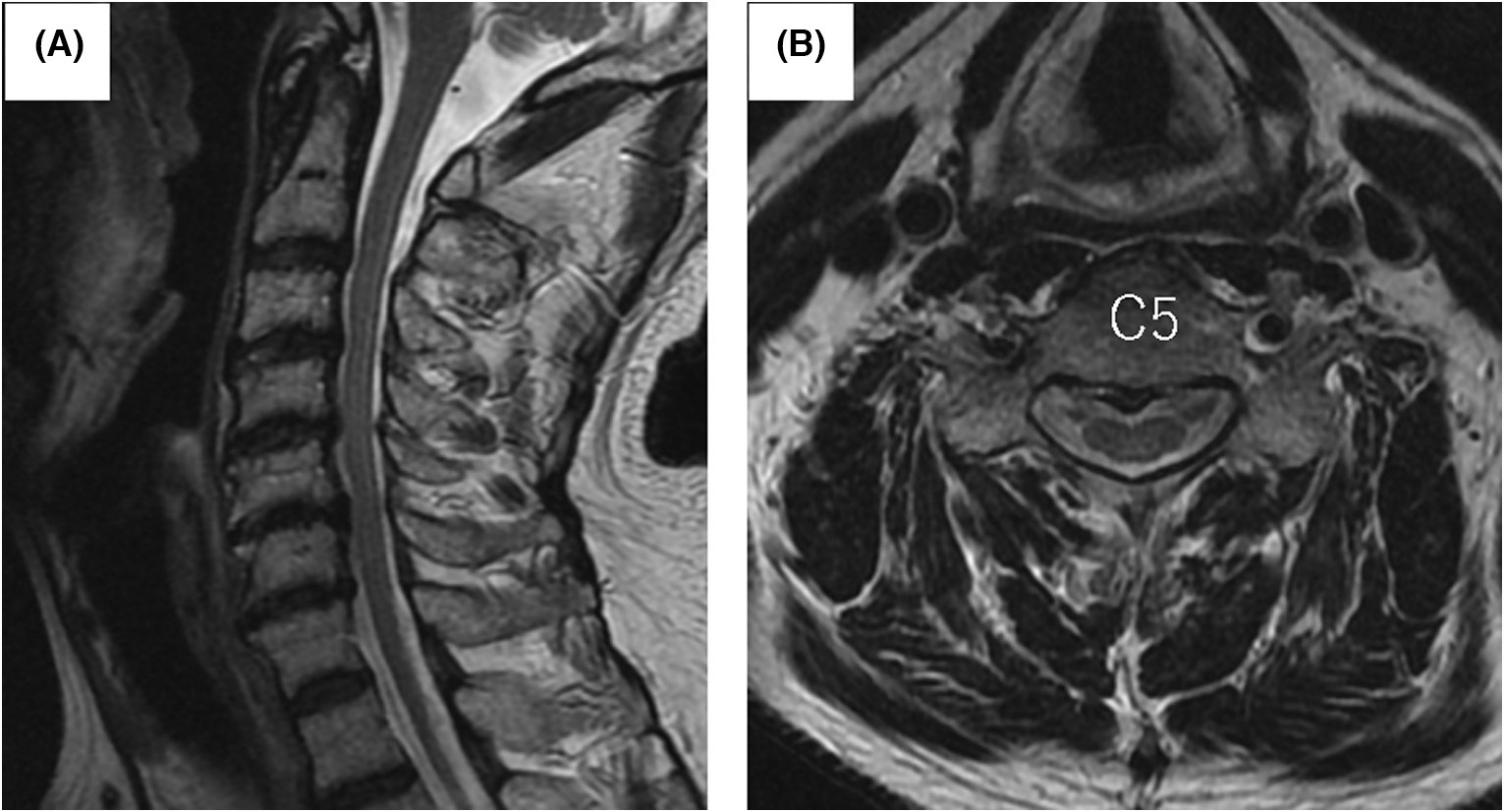
Magnetic resonance imaging of the cervical spine 6 months after treatment with subacute combined degeneration. Hyperintense signal changes in the spinal cord disappeared on both sagittal (A) and axial (B) MRI.

As for imaging findings, follow‐up MRI taken 6 months after the treatment for SCD showed the disappearance of hyperintensity in the spinal cord (Figure 3).

Later interviews revealed that he had a taste disorder (hypersensitivity to spicy food) just after the previous antibiotic treatment, but it gradually improved within a few months after the treatment with SCD.

## 4 DISCUSSION

Vitamin B12 and folate are cofactors for methionine synthase, an enzyme synthesizing methionine from homocysteine, essential for many genomic and non‐genomic methylation reactions. Therefore, vitamin B12 is necessary for developing and initial myelination of the central nervous system and maintaining normal function. Demyelination of the dorsal and lateral columns of the spinal cord can occur with vitamin B12 deficiency. However, the underlying pathophysiology of demyelination remains unclear. In the animal model, pathological analysis reveals a “spongy degeneration” because of the loss of and swelling of myelin sheaths.^6^ The diagnosis of SCD is made by detecting vitamin B12 deficiency, generally with a measure of the serum vitamin B12 level.^1^

Here, we present a case of SCD diagnosed after the progression of megaloblastic anemia symptoms. Clinical symptoms in patients with SCD include neurological symptoms of the extremities, such as limb numbness, limb weakness, and gait disturbance.^2^ The sensory symptoms occur earlier than motor symptoms in SCD patients.^2^, ^7^ The initial symptoms are most commonly paresthesia in the hands and feet, as in this case.^8^ At the time the diagnosis of SCD was made, the progression of numbness in his hands was only mild and Romberg's sign was absent, so it was most likely in the early stages of neurological symptoms. However, we cannot deny the possibility of delayed diagnosis, considering that the anemia was quite advanced. Furthermore, it has been reported that the degree of neurological dysfunction is correlated with the severity of megaloblastic anemia with vitamin B12 deficiency, although the reason is unclear.^9^, ^10^ Since the patient in this case already had gait disturbance associated with pyogenic spondylitis in the past, the lower extremity symptoms as a manifestation of SCD may have been overlooked. Actually his gait disturbance was slightly improved after treatment for SCD, which may prove the involvement of SCD in his lower extremity symptoms.

Paralysis with pyogenic spondylitis occurs because of mechanical damage of the spinal cord caused by disrupting the infected spine and compression with an epidural abscess. However, demyelination of the spinal cord's cervical and thoracic dorsal and lateral columns causes neural damage with SCD.^9^ Although it may be difficult to distinguish accurately from compression myelopathy in clinical practice, profound sensory disturbance, such as ataxia while walking and the Romberg's sign, are the typical clinical symptoms of demyelination of the posterior funiculus causes. However, typical posterior cord symptoms such as Romberg's sign are not seen in all SCD cases, as in this case.^11^ MRI helps detect the degeneration of the spinal cord, demonstrating the inverted V‐shaped hyperintensity within the posterior funiculus on T2‐weighed imaging,^3^, ^4^, ^12^ while conventional MRI is reported to have very low sensitivity as a tool for the diagnosis of SCD.^13^ Another significant feature of MRI findings in patients with SCD is the absence of direct compression of the spinal cord, which is useful in the imaging differentiation of SCD from other conditions such as pyogenic spondylitis. As presented, SCD of the spinal cord mainly affects the cervical and upper thoracic cord.^4^ Extreme caution is warranted in patients with pre‐existing lower extremity symptoms presenting with new upper extremity symptoms, even if they are trivial.

Malabsorption is a common cause of vitamin B12 deficiency.^1^ While autoimmune gastritis such as pernicious anemia is the most common cause of severe vitamin B12 deficiency,^14^ various factors can contribute to mild malabsorption of vitamin B12.^15^, ^16^ The case had been diagnosed with atrophic gastritis several years ago during a routine medical checkup by gastroscopy. Additionally, this patient had been receiving oral antibiotics for pyogenic spondylitis for 6 months, which may have aggravated atrophic gastritis and recently induced the progression of vitamin B12 malabsorption with SCD. We should be cautious about prescribing oral antibiotics for a long period in patients with gastrointestinal disorders. Furthermore, he noticed a taste disorder (hypersensitivity to spicy food) during antibiotic therapy, which continued until his recent admission for SCD. Glossitis is a known side effect of antibiotics and peripheral symptoms of SCD.^1^ In this instance, we were unaware of his taste disorder, probably because of the minor symptoms. However, the peripheral symptoms of SCD should always be considered when examining patients.

This case was worthy considering because neurological symptoms associated with SCD were overlooked and anemia progressed by the time the diagnosis was made, although the treatment was successful. Additionally, it should be understood that patients with pre‐existing lower‐extremity impairment may have difficulty recognizing lower‐extremity symptoms such as SCD.

## AUTHOR CONTRIBUTIONS

**Yoshiki Shimokawa:** Data curation; software; writing— original draft. **Yuki Ishikawa:** Data curation; writing— original draft. **Teiki Okawa:** Data curation; investigation; supervision. **Mana Higashihara:** Data curation; investigation; supervision; validation. **Fumiaki Tokimura:** Supervision; validation. **Tsuyoshi Miyazaki:** Supervision; validation. **Kentaro Hayakawa:** Conceptualization; data curation; supervision; writing— original draft.

## FUNDING INFORMATION

Teijin Pharma Limited.

## CONFLICT OF INTEREST STATEMENT

The authors have declared that no conflict of interest exists.

## CONSENT

Written consent was taken from the patient for the publication of the case report and the images.

## Data Availability

Data sharing was not applicable to this article as no datasets were generated or analyzed during the current study.
